# Characterization of Genetic and Epigenetic Variation in Sperm and Red Blood Cells from Adult Hatchery and Natural-Origin Steelhead, *Oncorhynchus mykiss*

**DOI:** 10.1534/g3.118.200458

**Published:** 2018-10-01

**Authors:** Mackenzie R. Gavery, Krista M. Nichols, Giles W. Goetz, Mollie A. Middleton, Penny Swanson

**Affiliations:** * School of Aquatic and Fishery Sciences, University of Washington, Seattle, WA 98105; † Conservation Biology Division; ‡ Environmental and Fisheries Sciences Division, Northwest Fisheries Science Center, National Marine Fisheries Service, NOAA, 2725 Montlake Blvd East, Seattle, WA 98112

**Keywords:** steelhead, epigenetics, DNA methylation, hatchery, genetic variation

## Abstract

While the goal of most conservation hatchery programs is to produce fish that are genetically and phenotypically indistinguishable from the wild stocks they aim to restore, there is considerable evidence that salmon and steelhead reared in hatcheries differ from wild fish in phenotypic traits related to fitness. Some evidence suggests that these phenotypic differences have a genetic basis (*e.g.*, domestication selection) but another likely mechanism that remains largely unexplored is that differences between hatchery and wild populations arise as a result of environmentally-induced heritable epigenetic change. As a first step toward understanding the potential contribution of these two possible mechanisms, we describe genetic and epigenetic variation in hatchery and natural-origin adult steelhead, *Oncorhynchus mykiss*, from the Methow River, WA. Our main objectives were to determine if hatchery and natural-origin fish could be distinguished genetically and whether differences in epigenetic programming (DNA methylation) in somatic and germ cells could be detected between the two groups. Genetic analysis of 72 fish using 936 SNPs generated by Restriction Site Associated DNA Sequencing (RAD-Seq) did not reveal differentiation between hatchery and natural-origin fish at a population level. We performed Reduced Representation Bisulfite Sequencing (RRBS) on a subset of 10 hatchery and 10 natural-origin fish and report the first genome-wide characterization of somatic (red blood cells (RBCs)) and germ line (sperm) derived DNA methylomes in a salmonid, from which we identified considerable tissue-specific methylation. We identified 85 differentially methylated regions (DMRs) in RBCs and 108 DMRs in sperm of steelhead reared for their first year in a hatchery environment compared to those reared in the wild. This work provides support that epigenetic mechanisms may serve as a link between hatchery rearing and adult phenotype in steelhead; furthermore, DMRs identified in germ cells (sperm) highlight the potential for these changes to be passed on to future generations.

Hatchery programs are used to produce fish for aquaculture, recreational fishing and conservation. In salmon and steelhead specifically, hatcheries are used extensively to supplement commercial harvest as well as to aid recovery of threatened or endangered populations (Naish *et al.* 2008). While conservation hatchery programs intend to produce fish that are genetically and phenotypically indistinguishable from the wild stocks they aim to restore, this has proven difficult to achieve. There is considerable evidence that salmon and steelhead reared in hatcheries differ from wild fish in phenotypic traits related to fitness even when wild fish are incorporated as broodstock (Frankham 2008, Fraser 2008). A number of studies have shown that hatchery fish exhibit reduced reproductive success relative to wild counterparts when spawning in the wild (Christie *et al.* 2014, Ford *et al.* 2016), and the loss of fitness in steelhead can occur rapidly; within one or two generations in the hatchery (Araki *et al.* 2007a, Araki *et al.* 2007b). Possible mechanisms of fitness loss include hatchery-induced selection (*i.e.*, domestication selection), and/or environmentally-induced epigenetic changes that are heritable across generations. Evidence for a heritable basis for fitness loss in steelhead comes from studies showing that hatchery fish do not perform as well as wild fish when raised in a common environment (Reisenbichler *et al.* 2004, Christie *et al.* 2012) but, this does not preclude other mechanisms, such as environmentally-induced epigenetic change. Very recently, it was reported that juvenile offspring of hatchery and natural-origin steelhead vary at the molecular level (*i.e.*, gene expression differences; Christie *et al.* 2016), but to what extent these differences contribute to adult phenotype, and whether the basis is heritable genetic or epigenetic change, is still uncertain. The idea that the early-rearing environment may induce persistent or heritable epigenetic change in hatchery fish has often been suggested (*e.g.*, Browman 1989, Jonsson and Jonsson 2014, Christie *et al.* 2012), but has yet to be directly tested. A previous study failed to identify global differences in DNA methylation between hatchery and natural-origin adult steelhead (Blouin *et al.* 2010), however this is not surprising as the methods used did not have the resolution to detect fine scale differences. More recently, a genome-wide study comparing DNA methylation patterns between hatchery and natural-origin coho salmon (*Oncorhynchus kisutch*) provided evidence that captive-rearing induced parallel changes in DNA methylation in the muscle of juvenile coho in two different river systems (Le Luyer *et al.* 2017). This is the first study linking hatchery-rearing and DNA methylation changes, however, the question remains if these DNA methylation changes in juveniles are reversible or persist in tissues of adults.

DNA methylation, along with other fundamental epigenetic marks such as histone modifications, can regulate genome accessibility and chromatin structure, which in turn can affect transcriptional activity (Bird 2002). In addition to regulating many cellular processes such as the temporal and spatial regulation of gene expression (Okano *et al.* 1999, Zhang *et al.* 2006) and promoting genome stability (Slotkin and Martienssen 2007), DNA methylation also has an important role in regulating cellular responses to environmental cues (Fraga *et al.* 2005). As such, variation in DNA methylation arising from environmental signals can have phenotypic consequences (Feil and Fraga 2012). For example, nutrition (Dolinoy *et al.* 2006), exposure to toxins (Dolinoy *et al.* 2007), and photoperiod (Azzi *et al.* 2014) in mammals have all been associated with changes in DNA methylation and concomitant changes in phenotype. Fish have been less studied, but nevertheless show similar environmental sensitivity in DNA methylation patterns (Wang *et al.* 2009, Strömqvist *et al.* 2010, Campos *et al.* 2013, Artemov *et al.* 2017).

Because it is mitotically stable (Bird 2002), DNA methylation provides a mechanism whereby early-environmental exposures can have persistent phenotypic effects in an individual (Weaver *et al.* 2004, Dolinoy *et al.* 2006, Heijmans *et al.* 2008). Early embryonic development is a particularly sensitive window for epigenetic changes to become fixed in an organism, as extensive DNA methylation remodeling occurs during cell differentiation and organ development in mammals (Meissner *et al.* 2008). In fish, there are numerous examples of demonstrating that environment during early life history stages can affect adult phenotype (reviewed by Jonsson and Jonsson 2014), but the underlying molecular mechanisms remain unclear. Evidence that DNA methylation during early development has lasting effects on adult phenotypes in fish comes from studies of temperature dependent sex determination (Navarro-Martín *et al.* 2011, Shao *et al.* 2014). For example, in sea bass (*Dicentrarchus labrax*) exposure to high temperature in early development was associated with increased DNA methylation in the promoter of the aromatase gene (*cyp19a1a*) and a higher proportion of phenotypic males (Navarro-Martín *et al.* 2011).

Environmentally-induced DNA methylation changes can also be meiotically stable in the germ line and passed to subsequent generations, which is referred to as transgenerational epigenetic inheritance (Guerrero-Bosagna *et al.* 2010, Manikkam *et al.* 2012). In order for DNA methylation change to be transgenerational, DNA methylation changes must occur in the gametes and avoid reprogramming or erasure in the embryo. As such, transgenerational epigenetic inheritance through DNA methylation is rare in mammals, which undergo extensive DNA methylation reprogramming (Daxinger and Whitelaw 2012). However, DNA methylation reprogramming strategies vary among vertebrates (Ci and Liu 2015). In zebrafish, the maternal methylome undergoes extensive resetting in early development, while the paternal methylome is stably inherited, such that at the time of zygotic gene activation the embryonic methylome resembles that of the father (Potok *et al.* 2013, Jiang *et al.* 2013). These findings raise questions about the potential for environmentally-induced, transgenerational epigenetic change in fish compared to mammals. Among the limited evidence in fish for environmentally-induced transgenerational epigenetic inheritance, DNA methylation is involved in environmental sex determination in half-smooth tongue sole (*Cynoglossus semilaevis*), and global methylation patterns are inherited by F1 ‘pseudomale’ offspring generated by crosses between temperature-induced sex-reversed ‘pseudomales’ and normal females (Chen *et al.* 2014, Shao *et al.* 2014). These data highlight the potential importance of DNA methylation in fish, and the clear need for more detailed studies on the effects of environmentally-induced epigenetic changes in a wider range of ecologically and economically important species such as salmonids.

Although DNA methylation is not well characterized in salmonids there is increasing evidence suggesting epigenetic mechanisms (*i.e.*, changes in DNA methylation) are associated with variation in life history phenotypes including early male maturation (Morán and Pérez-Figueroa 2011), smoltification (Morán *et al.* 2013), anadromy (Baerwald *et al.* 2015) and growth potential (Burgerhout *et al.*, 2017). Although the data are limited, there are some reasons to believe that DNA methylation may play a role in mediating the altered phenotypes (*i.e.*, reduced reproductive success) observed in hatchery-reared salmonids. Considering that, 1) hatchery reared salmonids encounter manipulated environmental parameters during critical windows in development when alterations in DNA methylation can become fixed in an organism, 2) differential DNA methylation has been observed in juvenile hatchery-reared coho salmon (Le Luyer *et al.* 2017), 3), DNA methylation plays an important role in mediating reproductive phenotypes in some fish species (Navarro-Martín *et al.* 2011, Shao *et al.* 2014), and 4) DNA methylation patterns are associated with various life history phenotypes in salmonids (Morán and Pérez-Figueroa 2011, Morán *et al.* 2013, Baerwald *et al.* 2015, Burgerhout *et al.*, 2017), it is important to investigate the potential epigenetic impacts of early rearing environment on adult hatchery-produced steelhead.

In this study, we evaluated genetic and epigenetic (DNA methylation) differentiation between hatchery and naturally spawned adult steelhead (*Oncorhynchus mykiss*) from the Methow River, WA. We utilized Restriction Site Associated DNA Sequencing (RAD-Seq) and reduced representation bisulfite sequencing (RRBS), to assess genetic and epigenetic variation respectively. In this study, DNA methylation was analyzed in both somatic (red blood cells (RBCs)) and germline (sperm) cell-types of adult steelhead. While any DNA methylation changes observed in somatic cells may affect the phenotype of the organism itself, changes in DNA methylation in the germline of hatchery fish have the potential to be passed to the next generation. The goal of this work is to describe the underlying genetic and epigenetic variation between hatchery and natural-origin adult steelhead in the Methow River as a first step toward understanding the possible mechanisms of observed fitness loss for wild steelhead after a single generation of rearing in the hatchery.

## Materials and Methods

### Fish and sample collection

Natural and hatchery-origin adult steelhead returning to the Methow River in summer 2013 and captured in late winter and spring 2014 were used in this study. Methow River summer-run steelhead are part of an Upper Columbia River evolutionary significant unit (ESU) of West Coast steelhead populations currently listed as threatened under the Endangered Species Act. Since the late 1960s this population has been influenced by releases of large numbers of hatchery fish that can interbreed with wild fish. Consequently, there is no distinct wild Methow River steelhead population free of genetic influence from artificial breeding and selection that occurs in the hatchery. Therefore, the term natural-origin is used to refer to a fish that was an offspring of fish spawning in the wild and lived its entire life in the wild, while a hatchery fish refers to a fish generated through artificial crosses and reared through juvenile stages in a hatchery (approximately 1 year) prior to being released into the wild.

Hatchery fish generated from hatchery by natural–origin crosses by the Washington Department of Fish and Wildlife (WDFW) Wells Hatchery (WH, Pateros, WA) and reared at either WH or the Winthrop National Fish Hatchery (WNFH, Winthrop, WA) and released as yearlings were used in this study. Adults returning to the Methow River were collected by hook and line by U.S. Fish and Wildlife Service (USFWS) staff and volunteers during February –April 2014 and held at either the WH or WNFH adult holding facilities until spawning. Hatchery fish are all ‘marked’ in this system, identifiable by adipose fin clip and/or presence of coded-wire tags in the snout. All unmarked fish were considered natural-origin fish (*i.e.*, fish spending at least one generation in the wild). Scale patterns were used to age all adults and determine freshwater residence time (Bernard and Myers 1996; data kindly provided by USFWS and WDFW). The ages of hatchery and natural-origin fish used in this study ranged from 3-4 and 4-5 years, respectively. Age differences between hatchery and natural-origin adults returning in a given year to the Methow River arise because natural-origin fish generally have longer residence in fresh water before migrating to sea than hatchery fish, which are grown to produce yearling smolts (Berejikian *et al.* 2012). Metadata for individual fish can be found in Supplemental Material, Table S1.

Prior to sample collection, all hatchery-origin fish (both sexes) and natural-origin males were selected for spawning and killed by hatchery staff. Natural-origin females were anesthetized and live spawned by USFWS and Yakama Nation biologists at the WNFH for transfer to the Upper Columbia Kelt Reconditioning Program. Milt (fish semen) was expressed by gentle abdominal pressure, collected into plastic bags and subsamples (0.1-0.3 ml) were aliquoted into each of three cryovials and frozen in liquid nitrogen. After gamete collection, whole blood (approximately 1.0 ml) was collected from the caudal vasculature using 21-gauge needles and heparinized 3cc syringes, and transferred into 1.5 ml microfuge tubes on ice. Prior to centrifugation, aliquots of blood were transferred to 0.4 ml polypropylene microfuge tubes and centrifuged at 10,000 × g for 6 min. Red blood cells (RBCs) were harvested by cutting the tip of tube with a new single-edged razor blade well below the plasma and buffy coat layer, transferred to cryovials using a micropipette, and frozen in liquid nitrogen. All samples were stored at -80° until analysis.

Red blood cells from a total of 79 fish (hatchery: 19 males and 20 females; natural origin: 19 males and 21 females) were used for the genetic analysis. The hatchery group included both WH (n = 24) and WNFH (n = 15) fish. Samples of sperm and RBCs from a subset of the male fish, 10 natural (age 5, n = 4; age 4, n = 6), and 10 hatchery (WH age 3, n = 6; WH age 4, n = 2; WNFH age 4, n = 2), were used for reduced-representation bisulfite sequencing (RRBS) analysis. These cell-types were selected for two reasons. First, both are composed of a single cell-type, eliminating any confounding factors due to differential methylation in mixed cell-types. Second, they represent both a somatic (RBC) and a germ line (sperm) derived cell-type.

### DNA isolation

Genomic DNA was isolated using the DNeasy Blood and Tissue Kit (Qiagen, Valencia, CA) following the protocol for animal blood with nucleated erythrocytes (for RBCs) or the modification for DNA isolation from sperm (for sperm). The Qubit dsDNA broad-range assay Kit (Qiagen) was used for DNA quantification.

### Restriction Site Associated DNA Sequencing (RAD-Seq) library preparation and analysis

DNA isolated from RBCs was used for RAD-Seq. Samples were prepared for RAD-Seq as described by Miller *et al.* (2007). Briefly, DNA was digested with *SbfI*, and then uniquely barcoded for library preparation. Library preparation was performed using the KAPA Low Throughput Library Prep Kit for Illumina platforms (KAPA Biosystems, Wilmington MA). Barcoded samples were divided into three libraries with an equal number of individuals per library. Each library was sequenced for 100 cycles in a single lane of a single-read flow cell on an Illumina HiSeq 2500 (University of Oregon, Genomics and Cell Characterization Core Facility).

Stacks v. 1.23 (Catchen *et al.* 2011) was used to process the RAD-Seq data. Reads produced from each lane of sequencing were first trimmed to 85 bases, quality filtered (default parameters were used), and de-multiplexed according to sample barcodes using *process_radtags*. Reads were aligned to the *O. mykiss* reference genome scaffolds (Berthelot *et al.* 2014) using bowtie 1.1.1 (parameters: *-n 3*, *-k 10,–best*). Unique stacks of possible alleles in each individual were grouped using *pstacks*, using a bounded single nucleotide polymorphism (SNP) model that bounded sequencing error between 0.001 and 0.01, and all other parameters at the default value, except for the minimum depth of coverage to create a stack within an individual (-m parameter), which was set to 3. Twenty individuals, 10 hatchery origin and 10 natural origin, with total filtered reads between 2.5 and 3.5 million, were used to construct the catalog in *cstacks*, with default parameters. All samples were aligned to the catalog using *sstacks* with default parameters. Genotypes were called for the first SNP in each locus and scored for each locus in each individual with a minimum depth of sequencing of 10 reads per locus. Seven individuals that had fewer than 75% of loci genotyped were removed from downstream analyses. Genotypes were further filtered to remove loci with: 1) a global minor allele frequency of less than 0.05, 2) greater than 80% observed heterozygosity to remove likely paralogous sequence variants, and 3) any missing data in a single individual (*i.e.*, included only genotypes sequenced in 100% of the individuals). Filtering steps were conducted using a combination of *populations* in Stacks and vcftools 0.1.12b (Danecek *et al.* 2011). A Principal Component Analysis (PCA) was performed on centered and scaled allele frequencies using the R package *adegenet* 1.4-2 (Jombart 2008) to evaluate global genetic diversity among genotypes. Genepop 4.5.1 (Raymond and Rousset 1995) was used to test for genotypic differentiation between hatchery and natural-origin fish using Fisher’s exact test. LOSITAN (Antao *et al.* 2008) was used to identify outlier SNPs that may be candidates for selection. LOSITAN was run with the options to calculate the ‘neutral’ mean F_ST_ from a candidate set of neutral loci determined from an initial run within LOSITAN, and to ‘force mean F_ST_’ allowing the approximation of the average simulated F_ST_ to the average value from the empirical data.

### RRBS library preparation and data analysis

Reduced representation bisulfite sequencing was performed on sperm and RBC DNA from 10 hatchery and 10 natural-origin fish. Samples were prepared for RRBS by digesting 3-5 μg of genomic DNA with *Msp*I restriction enzyme overnight at 37°. Digested DNA was purified using the MinElute PCR Purification kit (Qiagen) and 100-300 bp fragments were selected by gel extraction. Excised fragments were purified (MinElute PCR purification Kit, gel extraction protocol) and subjected to two rounds of bisulfite conversion using the MethylAmp Bisulfite Kit (Epigentek, Farmingdale NY). Libraries were prepared with 50-150 ng of bisulfite-treated DNA using the EpiNext Post-Bisulfite DNA library Preparation Kit (Epigentek) with barcoded adapters. Libraries were pooled (5-10 individuals per library) and sequenced for 100 cycles in 1-3 lanes (targeting 25 million/reads per individual) of a single-read flow cell on an Illumina HiSeq 2500 (University of Oregon, Genomics and Cell Characterization Core Facility).

Sequencing reads were quality and adapter trimmed using *TrimGalore!* (http://www.bioinformatics.babraham.ac.uk/projects/trim_galore) a wrapper for the publicly available trimming tool *cutadapt* (Martin 2011) and FastQC (Andrews 2010). TrimGalore! (v0.4.0) was run with default parameters and the additional RRBS specific options *–rrbs* and *–non-directional*. Trimmed reads were aligned to the *O. mykiss* reference genome scaffolds (Berthelot *et al.* 2014) with the bisulfite mapping tool Bismark v.14.3 (Krueger and Andrews 2011). Bismark used Bowtie2 for mapping ‘post-bisulfite adapter tagged libraries’ (option: *–pbat*) with the function for minimum score alignment set to allow approximately 3 mismatches per 100 bp read (option: *–score_min L,0,-0.2*). Count data for methylated and unmethylated reads were extracted using the *Bismark methylation extractor* script for downstream analysis.

Tests for differential methylation were performed in three separate comparisons: 1) RBC compared to sperm, regardless of origin (*i.e.*, cell-type specific methylation), 2) hatchery compared to natural-origin fish in RBCs (*i.e.*, RBC origin-specific methylation), 3) hatchery compared to natural origin in sperm (*i.e.*, sperm origin-specific methylation). The focus on differentially methylated DNA regions (DMRs) is based on findings that functionally relevant changes in methylation are typically associated with genomic regions rather than single CpG sites (Lister *et al.* 2009, Hansen *et al.* 2011). The R package *methylKit* (Akalin *et al.* 2012) was used to identify DMRs using 100 base pair non-overlapping tiling windows with a minimum of one CpG and no less than 20x total CpG coverage per tile in at least 14 of 20 samples for cell-type specific methylation and seven of 10 individuals for origin-specific methylation. Methylation differences were determined in *methylKit* using logistic regression and reported as the difference in average methylation across all CpGs in the tile (Akalin *et al.* 2012). Because hatchery fish used in this study were on average one year younger than the natural origin fish (hatchery fish spend only one year in freshwater), age was included as a model covariate for the origin-specific analyses. Significant DMRs included those with ≥ 20% difference in methylation between groups at the default qvalue of ≤ 0.01 (MethylKit uses a sliding linear model (SLIM) (Wang *et al.* 2011) for multiple testing correction). Visualization of DMRs across individuals was accomplished using the heatmap.2 function in the R package *gplots* (Warnes 2011). Hierarchical clustering of rows and columns was performed using Ward’s linkage method on Euclidean distances.

All regions analyzed, including DMRs, were annotated to genes using predicted gene models for the *O. mykiss* genome (Berthelot *et al.* 2014). DMRs overlapping genes and their putative regulatory domains (defined here as within 10kb of the transcription start site or transcription end site) were identified using the software BEDTools (Quinlan and Hall 2010). This annotation window was selected to include both proximal promoters as well as distal regulatory regions that may be epigenetically regulated (*e.g.*, Birshtein 2014, Libertini *et al.* 2015). Genes were aligned to the UniProtKB/Swiss-Prot database (http://uniprot.org) in order to determine homology to known protein sequences. Alignments were made using the BLAST algorithm (Altschul *et al.* 1990) (blastx with 1e-10 e-value cutoff). Associated gene ontology (GO) terms were assigned from the Gene Ontology dataset (Gene Ontology database: http://www.geneontology.org), and GOSlim terms based on the MGI GO Slim database (URL: http://www.informatics.jax.org). Regions not associated with genes were annotated for potential association with transposable elements using a genomic feature track associated with the *O. mykiss* reference genome (Berthelot *et al.* 2014, http://www.genoscope.cns.fr/trout/data/) in BEDTools. In order to determine the proportion of regions associated with CpG islands, the EMBOSS tool *Cpgplot* (Rice *et al.* 2000) was utilized with default settings (window size 100 bp, minimum length 200bp, minimum observed to expected ratio C plus G to CpG 0.6, minimum percentage GC 50%). CpG island shores were defined as genomic regions within +/− 2kb of CpG islands and CpG shelves were defined as regions either 2kb upstream or downstream of shores. DMRs were annotated to CpG island features using BEDTools and those regions not annotated to a CpG island feature were defined as being ‘open sea’. Functional enrichment of GO terms and enrichment for CpG islands associated with DMRs was performed using corrected p-values from a two-sided Fisher’s Exact Test in R. A minimum number of hits (*i.e.*, > 4) was required for a GO term to be considered for enrichment. Adjusted p-values (Benjamini-Hochberg (BH)) of < 0.01 were considered significant. Origin-specific DMRs were also mapped to chromosomes based on their scaffold locations using the AGP file provided by Berthelot *et al.* (2014) and a custom perl script.

### Data Availability

File S1 contains RAD - genome alignment and LD analysis. Table S1 contains metadata for individual fish used in RAD and RRBS. Table S2 contains RAD loci sequences. Table S3 contains the RAD genpop file. Table S4 contains RRBS read count and mapping information. Table S5 contains annotation tables of DMRs. RAD and RRBS sequence data are available in the NCBI SRA database under the BioProject PRJNA325786. Supplemental material available at Figshare: https://doi.org/10.25387/g3.7152671.

## Results

### Genetic analysis (RAD-Seq)

**Figure 1:**
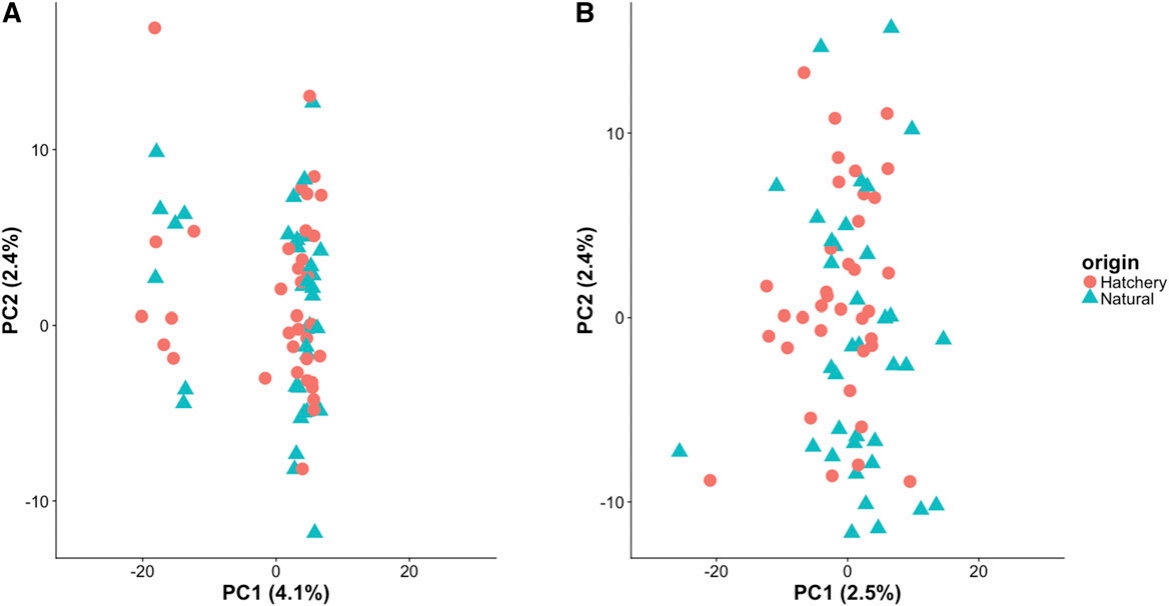
PCA ordination of RAD-Seq data. Individual hatchery and natural-origin fish are plotted according the first 2 principal components (PC1 and PC2) using 936 SNPs (A). The PCA was repeated after removing a group of markers in very high LD using 907 SNPs (B).

Genetic analysis of Methow River steelhead was performed to evaluate whether hatchery and natural-origin fish were genetically differentiated. RAD-Seq on 3 lanes of Illumina HiSeq generated 3.2 million reads per individual on average. A total of 29,580 candidate SNPs were identified and 936 SNPs remained (Tables S2 and S3) after filtering for global minor allele frequency (13,075 SNPs), heterozygosity (42 SNPs) and missing data (15,527 SNPs). Tests for global genotypic differentiation between hatchery and natural-origin fish were not significant (P-value: 1.00). F_ST_ outlier analysis, performed to identify potential loci under selection between the hatchery and natural-origin groups, identified only a single outlier at FDR 0.05 (marker 48889 Table S2). Initially, principal component analysis (PCA) of RAD-Seq data in the hatchery and natural-origin fish revealed the presence of two clusters separated along the PC1 axis (Figure 1A). However, these clusters did not correspond to the hatchery and natural-origin samples. Further analysis identified a group of markers heavily loading on the PC1 axis that were in high linkage disequilibrium (LD); a majority of which were located on chromosome 5 (see Supplemental File 1). Because this highly correlated group of SNPs has the potential to distort the PCA by giving higher weight to the redundant markers, they were removed and a second PCA analysis was performed using a final filtered set of 907 loci. Plotting of the first two principal components from the PCA did not show any clear genetic differences between hatchery (n= 36) and natural-origin (n = 36) steelhead from the Methow River (Figure 1B), and there was no obvious clustering according to age or sex of the fish (data not shown).

### Cell-type specific DNA methylation patterns

Reduced representation bisulfite sequencing of RBCs and sperm from both hatchery and natural-origin fish yielded an average of 38 million total reads per individual (34 million after quality trimming; Table S4). The average mapping efficiency to the *O. mykiss* genome was 39%. A proportion of the total mapped reads (approximately 22%) mapped to more than one location in the genome. Only reads with unique mappings were used for further analysis. On average, 643,318 CpG dinucleotides were sequenced at 10x coverage per individual (range: 303,229 – 992,591). The total proportion of methylated cytosines in a CpG context was 94% in sperm and 88% in RBCs. The proportion of non-CpG methylation was very low for both tissues (0.9% and 0.6% on average in sperm and RBC respectively).

A total of 112,247 non-overlapping 100bp regions (average 5 CpG per region) met the coverage criteria for analysis of tissue/cell-specific methylation. Regardless of fish origin, CpG dinucleotides in the reduced representation genome of *O. mykiss* were heavily methylated. Although the percent methylation of a particular 100 bp region is an average methylation of all CpGs in that region, in this analysis 96% and 88% of the regions in the sperm and RBCs, respectively, are fully methylated (>80% methylated) (Table 1). A majority of regions were hyper-methylated in both cell-types, but a small number of regions (1196) were hypo-methylated (*i.e.*, ≥25% methylation) in both cell-types (Table S5.4).

**Table 1:** Distribution of percent methylation per region for RBCs and sperm

Percent Methylation	RBC	Sperm
≥80%	88%	96%
20.1–79.9%	11%	2%
≤20%	1%	1%

**Figure 2:**
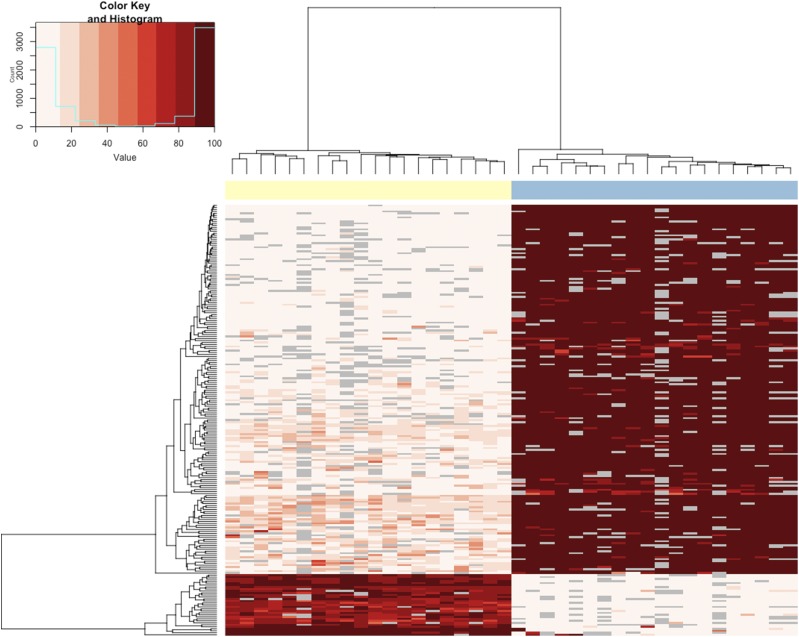
Hierarchical clustering of cell-type specific DMRs (217 tDMRs with >75% difference in methylation). Each column represents a cell-type from an individual fish. The cell-types are identified by color (RBCs = yellow, sperm = blue) at the top of the column. Each row represents a DMR. The heatmap depicts percent methylation for each 100bp region for each individual (n = 20 RBC, n = 20 sperm) with the darkest red indicating 100% methylation and the lightest indicating 0% methylation. The regions that did not meet the coverage cutoff for a particular individual are represented by gray boxes.

Using RRBS data from both a somatic (RBC) and germline (sperm) cell-types from the same individuals, we examined cell/tissue-specific differentially methylated regions (tDMRs) in addition to identifying origin-specific differences. We identified 3916 tDMRs between sperm and RBCs (Table S5.1); 95% of the tDMRs were hyper-methylated in sperm. Hierarchical cluster analysis of the most extreme tDMRs (≥75% difference in methylation between sperm and RBC) shows tight clustering within cell-types and highlights two clusters of tDMRs: one large cluster containing 190 tDMRs that were hypermethyated in sperm and a small cluster containing 33 tDMRs were hypo-methyated in sperm compared to RBCs (Figure 2).

**Figure 3:**
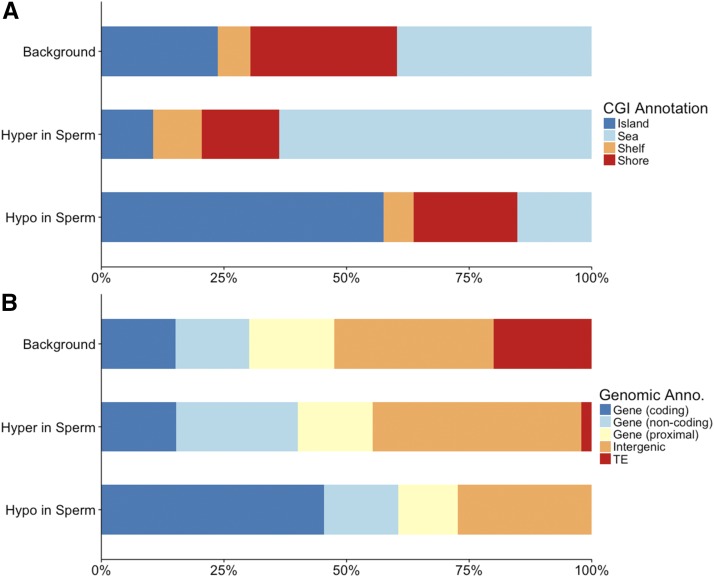
Annotation of tissue-specific DMRs (tDMRs) (Hypo in Sperm and Hyper in Sperm (compared to RBCs)) compared to the background of all 100 bp regions analyzed by RRBS (Background). Top panel (A) shows annotations relative to CG islands. Bottom panel (B) shows annotation relative to genes (coding regions (coding), intronic and untranslated regions (non-coding), within 10kb of a gene (proximal)), transposable elements (TE) and unannotated intergenic regions.

**Figure 4:**
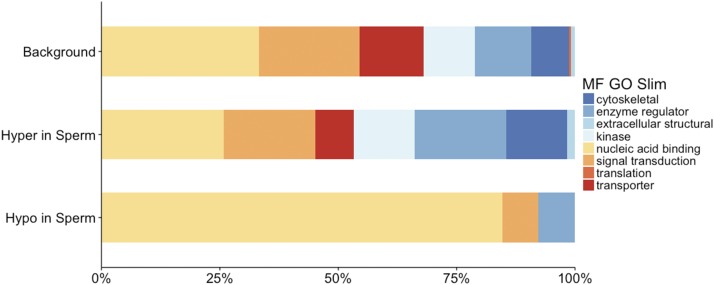
G0Slim Molecular Function annotation of tDMRs (Hypo in Sperm) and Hyper in Sperm (compared to RBCs)) compared to the background of all 100 bp regions analyzed by RRBS (Background).

Annotation of these DMRs highlights differences between tDMRs (>75% methylation difference between sperm and RBCs) when compared to annotation of the ‘background’ (*i.e.*, the 112,247 non-overlapping 100bp regions analyzed). In relationship to CpG islands (Figure 3A and Table S5.3), tDMRs that are hypo-methylated in sperm are enriched for CpG islands when compared to background (BH adjusted Fisher’s Exact Test, *P* = 1.3 × 10^−4^). In relationship to genomic features (Figure 3B and Table S5.2), tDMRs that are hypo-methylated in sperm are enriched for coding regions when compared to background (BH adjusted Fisher’s Exact Test, *P* = 1.6 × 10^−4^). Functional annotation of genes associated with tDMRs (Figure 4 and Table S5.3) revealed that tDMRs hypo-methylated in sperm are enriched in nucleic acid binding functions compared to background (BH adjusted Fisher’s Exact Test, *P* = 1.3 × 10^−3^). Interestingly, annotation to the gene level revealed a vast majority of the genes are homologous to transcription factors involved in early development (Table 2). Many of the regions that are hypo-methylated in both cell-types are also transcription factors (*i.e.*, most prevalent GO Slim term for molecular function is ‘nucleic acid binding’) (Table S5.4).

**Table 2:** Genes associated with tDMRs that are hypo-methylated in sperm

*O. mykiss* gene ID	UniProt ID	UniProt Gene Name	e-value
GSONMT00073496001	HXB3A_DANRE	Homeobox protein Hox-B3a	0
GSONMT00073495001	HXB1_CYPCA	Homeobox protein Hox-B1	3.00E-138
GSONMT00066870001	RPGF2_HUMAN	Rap guanine nucleotide exchange factor 2	0
GSONMT00065649001	ZN503_DANRE	Zinc finger protein 503	0
GSONMT00054294001	HXA2B_DANRE	Homeobox protein Hox-A2b	2.00E-162
GSONMT00054113001	PAF15_XENLA	PCNA-associated factor	3.00E-31
GSONMT00052579001	HXB3A_DANRE	Homeobox protein Hox-B3a	0
GSONMT00038401001	ZAR1_XENLA	Zygote arrest protein 1	2.00E-86
GSONMT00033437001	PO5F1_DANRE	POU domain, class 5, transcription factor 1	3.00E-175
GSONMT00033334001	HXA3A_TAKRU	Homeobox protein Hox-A3a	0
GSONMT00033304001	BMP15_SHEEP	Bone morphogenetic protein 15	3.00E-38
GSONMT00023217001	KCTD5_RAT	BTB/POZ domain-containing protein KCTD5	7.00E-126
GSONMT00019901001	ACINU_HUMAN	Apoptotic chromatin condensation inducer in the nucleus (Acinus)	2.00E-99
GSONMT00011735001	FOXE4_XENLA	Forkhead box protein E4	2.00E-94
GSONMT00011470001	HXA3A_TAKRU	Homeobox protein Hox-A3a	7.00E-174
GSONMT00009527001	IRX3_XENTR	Iroquois-class homeodomain protein irx-3	4.00E-56
GSONMT00004484001	SKDA1_HUMAN	SKI/DACH domain-containing protein 1	5.00E-69

### Hatchery and natural-origin-specific DNA methylation patterns

#### Red blood cells:

**Figure 5:**
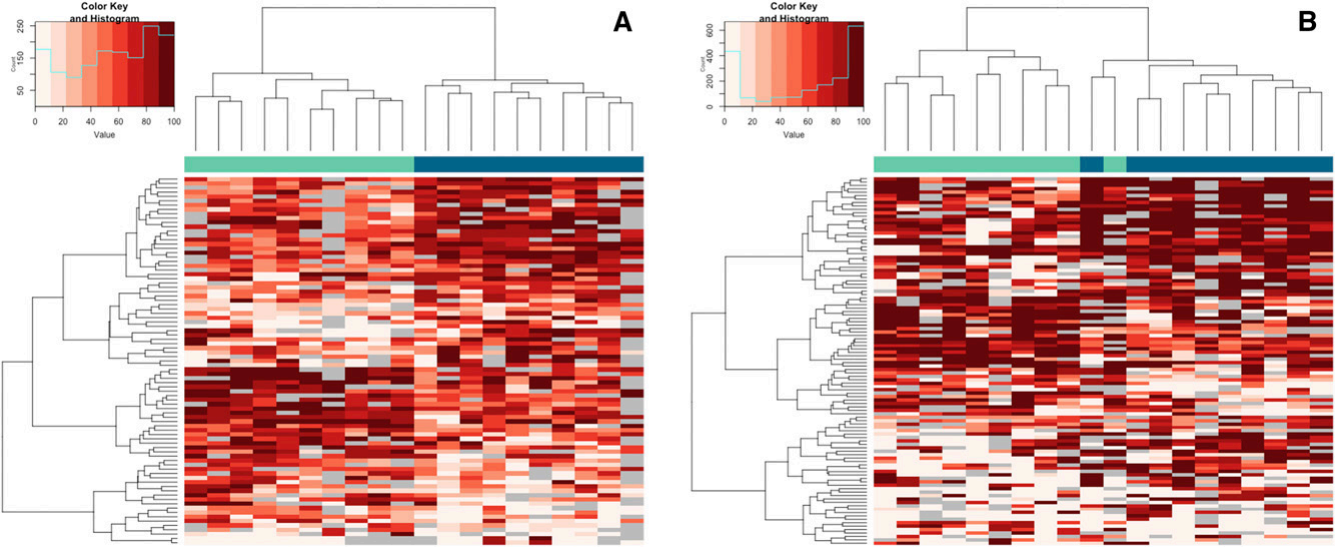
Hierarchical clustering of origin specific DMRs for RBCs (left panel) and sperm (right panel). Each column represents an individual. Fish origin is identified by color (natural = green, hatchery = blue) at the top of the column. Each row represents a DMR. The heatmap depicts percent methylation for each 100bp region for each individual (n = 10 hatchery origin, n = 10 natural origin) with the darkest red indicating 100% methylation and the lightest indicating 0% methylation. The regions that did not meet the coverage cutoff for a particular individual are represented by gray boxes.

**Figure 6:**
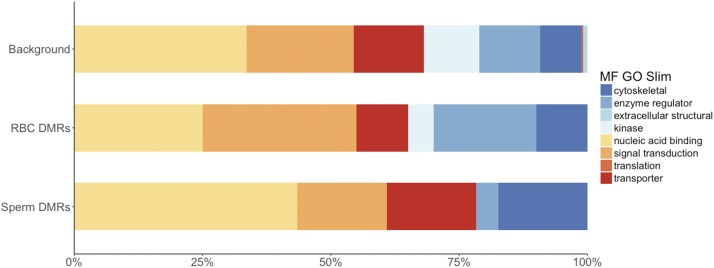
G0Slim Molecular Function annotation of origin-specific sperm and RBC DMRs compared to the background of all 100 bp regions analyzed by RRBS (Background).

**Figure 7:**
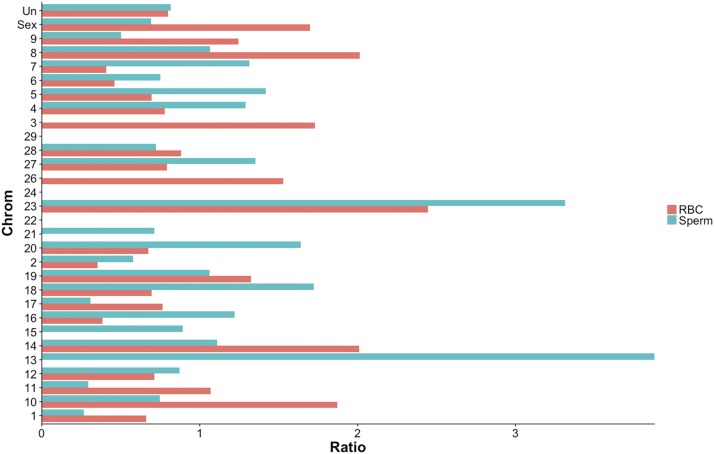
Number of origin-specific DMRs for RBCs and sperm per chromosome normalized by the expected number of DMRs per chromosome if the distribution of DMRs were completely random (*i.e.*, the expected number of DMRs per chromosome is a proportion of total DMRs relative to the total number of regions analyzed per chromosome). DMRs that could not be mapped to a chromosomal location (referred to as ‘ChrUn’ in the reference genome (Berthelot *et al.* 2014) are not included.

We identified 85 DMRs in the RBC methylomes between hatchery and natural-origin fish (Table S5.5). The difference in methylation was 20–46%, with almost an equal number being hyper- and hypo-methylated in hatchery compared to natural-origin fish. Individuals clustered according to origin based on the 85 DMRs (Figure 5A). Over half of the DMRs were associated with genes. Twenty-three DMRs were located within gene bodies (7 of these overlapped with coding regions), and another 23 DMRs were associated with putative regulatory regions of genes (*i.e.*, within 10 kb of a gene). When functionally annotated, the most common molecular function GO Slim terms for RBC DMRs were ‘signal transduction’ activity and ‘nucleic acid binding’ (Figure 6). Of the 39 DMRs that were not associated with genes, 13 of them mapped to transposable elements. Almost half of the origin-specific RBC DMRs were associated with either CpG islands (21%), shores (19%) or shelves (7%), the other half were not associated with any type of CpG island feature (*i.e.*, “open sea”). Forty-seven of the DMRs were mapped to known chromosomal locations. Almost all chromosomes contained at least one DMR. Chromosomes 23 had the highest number of DMRs relative to the number of regions analyzed, but no chromosome had a highly disproportionate number of DMRs (Figure 7).

#### Sperm:

There were 108 origin-specific DMRs identified in sperm (Table S5.6). DMRs were both hyper- (n = 66) and hypo-methylated (n = 42) in sperm of hatchery compared to natural-origin fish; the difference in methylation ranged from 20–66% percent. Individuals clustered according to origin based on the 108 DMRs, with the exception of a single natural-origin fish that clustered with the hatchery-origin group (Figure 5B). Twenty-five DMRs were located within gene bodies (23 of these overlapped with coding regions) and another 35 DMRs were associated with putative regulatory regions of genes (*i.e.*, within 10 kb of a gene). When functionally annotated, no molecular function GO Slim term for origin-specific sperm DMRs were significantly enriched, but ‘nucleic acid binding’ was the most common GO slim term (Figure 6). Of the 48 DMRs that were not associated with genes, 14 of them mapped to transposable elements. Sixty-one of the DMRs were mapped to known chromosomal locations. Almost all chromosomes contained at least one DMR. Chromosomes 13 and 23 had the highest number of DMRs relative to the total number of regions analyzed on that chromosome (Figure 7). Twenty of the origin-specific sperm DMRs overlapped with RBC DMRs.

## Discussion

Relative reproductive success studies have documented substantial fitness loss for wild steelhead after a single generation of rearing in the hatchery, but the relative contribution of genetic selection and/or environmentally-induced epigenetic changes passed through the germline remain unknown (Araki *et al.* 2008). Here, as a step toward understanding mechanisms of fitness loss, we describe genetic and epigenetic variation in adult hatchery and natural-origin steelhead. Our main objectives were to determine if hatchery and natural-origin fish from this stock could be distinguished genetically by examining SNPs across the entire genome, and whether differences in epigenetic programming (DNA methylation) in somatic and germ cells could be detected between the two groups. Genetic analysis using RAD-Seq did not reveal differences between the hatchery and natural fish at the population level. Nevertheless, we found significant differences in epigenetic programming in both somatic (RBCs) and germ cells (sperm). Using RRBS, we generated the first genome-wide characterization of somatic cell and germline derived DNA methylomes in a salmonid fish, from which we identified both cell-type specific and origin-specific methylation. Because hatchery fish experience similar environmental conditions as their wild conspecifics once they leave the hatchery, our results raise the possibility that these DNA methylation changes may have occurred during the first year in the hatchery and persisted into adulthood in the form of an ‘epigenetic memory’ of the hatchery environment. The idea that epigenetic mechanisms may serve as the link between early-environmental exposures and adult phenotypes (*i.e.*, ‘developmental programming’) has been previously suggested in fish (*e.g.*, Browman 1989, Jonsson and Jonsson 2014, Gavery and Roberts 2017).

### Genetic analysis

A genome wide analysis of genetic variation in hatchery and natural-origin fish captured from the Methow River was conducted to first establish whether these were distinct populations. The results from analyses using nearly 1000 RAD-Seq SNPs indicated no discernable genetic differences at the population level. We identified a single locus under selection at 0.05 FDR using a genome scan. One limitation of the approach is the use of reduced-representation technologies, and therefore we can’t rule out that selection hasn’t occurred in genomic regions that were not analyzed. Our results are consistent with previous genetic analyses of the Methow River steelhead population using microsatellite DNA (Blankenship *et al.* 2011). Moreover, the lack of genetic differentiation between hatchery and natural-origin fish is not surprising because the hatchery fish used in our study were generated from hatchery by natural-origin crosses at the WNFH and WH during 2010 and 2011. Furthermore, returning hatchery-origin steelhead spawn in the Methow River, and some unknown percentage of the natural-origin steelhead likely had hatchery-origin parents. The integration of natural origin Methow River adult steelhead into the hatchery breeding program and the likely natural spawning of hatchery fish in the Methow River over many decades has served to minimize genetic divergence between the hatchery and wild origin fish in this system, as demonstrated by our genetic data and analyses. Our results are consistent with both Waters *et al.* (2015) who found minimal genetic differentiation in an integrated hatchery line and Le Luyer *et al.* (2017) who found no genetic differentiation between hatchery and natural-origin coho salmon in two rivers using similar integrated hatchery approaches.

### Epigenetic analysis

In addition to genetic variation, epigenetic variation also has the potential to contribute to phenotypic differences between hatchery and wild steelhead. However, functional analysis of epigenetic mechanisms and variation in natural populations remains largely understudied. The increased availability of genomic resources and advancements in methods used to analyze DNA methylation are now allowing investigations of patterns and functions of DNA methylation in non-model organisms at high resolution and at a genome-wide scale. Here we used RRBS, which provides much higher sensitivity and resolution compared to methods such as methylation-sensitive amplified polymorphism (MSAP) (Li *et al.* 2008) that have been traditionally used to study DNA methylation in non-model organisms. For example, a previous study in Hood River steelhead failed to identify differences in DNA methylation between hatchery and wild fish (Blouin *et al.* 2010), but as the authors noted, the MSAP approach did not allow them to detect fine-scale differences. The RRBS approach does have limitations in that conclusions can’t be drawn about the portion of the genome that has not been analyzed as well as the inability of bisulfite sequencing technologies to discern a true C/T SNP from a methylation change. Results from RRBS used in the present study demonstrate that the steelhead genome is heavily methylated as in other vertebrates (Feng *et al.* 2010) and, like other fish species studied to date, methylation levels are higher than what is reported in mammals (Jabbari and Bernardi 2004, Chatterjee *et al.* 2013). Further, consistent with findings in zebrafish (*Danio rerio*) (Chatterjee *et al.* 2013), the distribution of genomic features analyzed using RRBS may not be as biased toward core CpG islands as it is in mammals and includes a greater proportion of island shores, which also have important regulatory functions (*e.g.*, Irizarry *et al.* 2009).

#### Cell-type specific DNA methylation patterns:

Initially we compared methylomes of sperm and RBCs to validate methodology and characterize germ-line specific DMRs. In both hatchery and natural-origin steelhead, a higher proportion of CpG sites were methylated in sperm compared to RBCs, which was expected because it is a characteristic feature of sperm DNA (Molaro *et al.* 2011, Potok *et al.* 2013, Jiang *et al.* 2013). The DNA methylation patterns in steelhead were cell-type specific and the majority of tDMRs were in CpG islands and shores, similar to what has been reported for mammals (Irizarry *et al.* 2009, Deaton *et al.* 2011, Davies *et al.* 2012). While most of the tDMRs were hyper-methylated in sperm, a small number of tDMRs were hypo-methylated compared to RBCs. Genes associated with hypo-methylated regions in steelhead sperm were almost exclusively those involved in cell and embryonic development (*e.g.*, *pou5F1* (oct 4), various *hox* genes, etc.), similar to findings in zebrafish (Potok *et al.* 2013) and mouse (Hammoud *et al.* 2009). While DNA methylation patterns in somatic cells are important for regulating gene expression and maintaining a cellular environment that is flexible to respond to environmental changes (Jaenisch and Bird 2003, Wan *et al.* 2015), the functions of the DNA methylation patterns are less clear in mature sperm, which contain various RNAs, but are transcriptionally silent (Grunewald *et al.* 2005). In mammals, it has been suggested that in addition to genetic information, the sperm transmits epigenetic information that may be involved in regulating early embryonic development (Hammoud *et al.* 2009). Recent studies in zebrafish suggest that the functional significance of sperm DNA methylation patterns in fish is to provide transcriptional competency to the early embryo, which ‘inherits’ the DNA methylation pattern in the sperm (Potok *et al.* 2013, Jiang *et al.* 2013). Since fish appear to lack imprinted genes (Hore *et al.* 2007), this may provide a mechanism for paternal specific effects on early embryonic phenotypes. Clearly, more research is needed to determine how the embryo is affected by parental DNA methylation patterns in fish, but it raises the possibility that environmentally induced changes in DNA methylation in sperm could be passed to offspring.

#### Hatchery and natural-origin-specific DNA methylation patterns:

Our finding of DMRs between hatchery and natural-origin fish for both RBCs and sperm suggests that early rearing environment may influence DNA methylation patterns in somatic and germ cells. Our results in adult hatchery-reared steelhead from a single river system are consistent with findings that hatchery-rearing induces parallel DNA methylation changes in juvenile coho salmon from two rivers (Le Luyer *et al.* 2017). The hatchery-specific DMRs in RBCs include both hyper- and hypo-methylated regions, are distributed across many chromosomes, and approximately half are associated with a functionally diverse set of genes. In fish, RBCs are transcriptionally active and, in addition to playing a major role in respiratory gas exchange, they participate in a variety of other processes including immune function, sugar transport, and calcium and redox homeostasis (Morera *et al.* 2011, Morera and Mackenzie 2011). The pattern of DNA methylation in mature RBCs reflects changes in methylation that occurred during differentiation of hematopoietic stem cells (HSCs) during embryonic development and differentiation of HSCs to mature RBCs (Davidson and Zon 2004), as well as changes associated with the dynamic nature of the mature RBC transcriptome as it responds to the environment and any inherited epigenetic variation. Although the mature RBCs sampled in our study contain RBCs of various ages, they would have been generated well after fish were released from the hatchery into habitat shared with wild/natural-origin fish. Thus, it is likely that the observed DMRs in RBCs from hatchery *vs.* natural-origin fish occurred as a result of differences in environment experienced in the first year of life. DNA methylation patterns have also been shown to correlate with early-rearing conditions in RBCs of adult hens (Pértille *et al.* 2017). While DNA methylation changes in RBCs could potentially alter gene transcription in the individual, these would not be passed on to future generations.

To establish whether there is potential for inter- or transgenerational inheritance of hatchery induced epigenetic changes through the germline we compared DNA methylation in sperm from hatchery and natural-origin fish and found significant differences across various genomic regions. Twenty of the sperm DMRs overlapped with regions identified as being differentially methylated in RBCs. In all cases, the direction of methylation change was the same for both cell types perhaps suggesting changes in methylation in these regions may be more general responses to hatchery-rearing. Although a proportion of the DMRs were common across cell types, most were unique to sperm. This is likely a reflection of the highly specialized characteristics and functions of DNA methylation in sperm compared to somatic cells. As described above, DNA methylation in sperm is important in the generation and maintenance of proper chromatin structure (Bourc’his and Bestor 2004, La Salle and Trasler 2006), but also may play an important role in regulation of genes in the early embryo (Hammoud *et al.* 2009, Potok *et al.* 2013, Jiang *et al.* 2013). In steelhead, we found that origin-specific sperm DMRs were associated with both hypo- and hyper-methylation of DNA and many of the regions were associated with genes. The most common molecular function for genes associated with origin-specific DMRs in sperm was nucleic acid binding. Interestingly, a majority of the genes hypo-methylated in sperm were also involved in nucleic acid binding, perhaps indicating that transcriptional regulators are preferentially sensitive to changes in DNA methylation in this cell-type. DNA methylation is particularly dynamic in developing germ cells and in mammals, a majority of the methylation marks are erased in primordial germ cells (PGCs) and reestablished in a sex specific manner during the differentiation of prospermatagonia (Seisenberger *et al.* 2012). There is increasing evidence that the developmental window coincident with these changes in germline methylation is a particularly sensitive period when chemical or nutritionally-induced changes in DNA methylation can have adverse effects on offspring (Ly *et al.* 2015). In rainbow trout, primordial germ cells migrate to the genital ridge and differentiate into spermatogonia or oogonia within the first few months of life, starting around the time of hatch (Devlin and Nagahama 2002). For hatchery fish, this sensitive window occurs while fish are exposed to many environmental variables that are different than what they would be exposed to in the wild (*e.g.*, temperature, water chemistry, diet, etc.). The differences in methylation observed here in sperm from hatchery *vs.* natural-origin fish are likely the result of changes in methylation that occurred during differentiation of prospermatagonia and became fixed in the germ cell lineage. It is still unclear from our study whether these changes in DNA methylation have functional consequences in the sperm and/or offspring. While there have been a considerable number of studies showing phenotypic differences in hatchery and wild fish (Araki *et al.* 2007a, Araki *et al.* 2007b, Christie *et al.* 2014, Christie *et al.* 2016), linkages to environmentally-induced epigenetic changes in adults have not been established. However, based on studies in mammalian systems, this could be an important mechanism linking early environment with adult phenotype with the potential for inter- and even transgenerational inheritance in hatchery fish.

Instances of environmentally-induced changes in DNA methylation patterns being passed through the male germline have been reported in mammals (Guerrero-Bosagna *et al.* 2010, Manikkam *et al.* 2012). Based on these findings, as well as increasing evidence that DNA methylation patterns in sperm may be important to the early embryo, paternal transmission of epigenetic information has been the topic of many recent reviews and perspectives (Rando 2012, Soubry *et al.* 2014, Casas and Vavouri 2014, Soubry 2015,). In fish, environmentally-induced transgenerational phenotypes have been documented (Corrales *et al.* 2014, Bhandari *et al.* 2015, Knecht *et al.* 2017), although the molecular mechanisms underlying these phenotypes have yet to be fully explored some evidence suggests that epigenetic mechanisms may be involved (Knecht *et al.* 2017). As mentioned previously, whole genome DNA methylation profiling in gametes and early developmental stages in zebrafish suggest that embryos inherit the sperm DNA methylation pattern, however the sperm DNA methylation pattern itself is not required as a ‘template’ suggesting that other molecular factors, such as small RNAs may be important for establishing proper DNA methylation patterns in embryos (Potok *et al.* 2013, Jiang *et al.* 2013). Together, these data highlight the potential of DNA methylation or other epigenetic marks to influence offspring phenotypes.

Considerable variation was observed in DNA methylation among individuals for both hatchery and natural-origin fish (Figure 5). Epigenetic diversity may have adaptive value to a population, as epigenetic variation has the potential to contribute to phenotypic variation in populations even in the absence of genetic variation (Jablonka and Raz 2009). Epigenetic diversity alone has been associated with increased niche width in clonal flowering yeast (Herrera *et al.* 2012) and increased productivity and stability of plants at a population level (Latzel *et al.* 2013). There are several factors that may contribute to the high degree of variation in DNA methylation patterns in this study. First, epigenetic variation could arise from natural epigenetic diversity in the population. Only a limited number of studies have addressed the range of epigenetic diversity in vertebrates, but some have suggested that it may be higher than genetic diversity (Schrey *et al.* 2012, Liu *et al.* 2015). Second, some variation in DNA methylation may be associated with genetic variation among individuals. Although hatchery and natural-origin fish could not be genetically distinguished at a population level, it is still possible that the methylation status of a particular CpG site could be influenced by the surrounding genetic sequence (Hellman and Chess 2010, Gertz *et al.* 2011). One limitation of this study is that there was little overlap between the RAD-Seq and RRBS loci to examine the hypothesis of underlying genetic variation promoting differences in the epigenome. These sources of variability highlight the challenges of causally associating changes in DNA methylation with phenotypes in a natural population. Studies using controlled genetic background and simulated ‘hatchery’ and ‘natural’ environments are currently underway to limit the effects of background genetic variation and age to directly address if early-rearing environment influences DNA methylation reprogramming in hatchery steelhead.

### Conclusion

In summary, our analysis of genome-wide patterns of genetic and epigenetic (DNA methylation) variation in hatchery and natural-origin Methow River steelhead confirm that hatchery fish in this stock are not genetically distinct from the natural population, yet considerable epigenetic variation was found. We provide the first fully characterized methylome of somatic and germline cell-types in a salmonid and find compelling evidence that early rearing environment may alter epigenetic programming in sperm. This work provides a foundation for future epigenetic studies in steelhead, and complements recent work aimed at exploring genetic mechanisms of fitness loss in hatchery reared steelhead. Alternative rearing regimes are being tested to evaluate effects on performance of hatchery-reared steelhead (*e.g.*, Berejikian *et al.* 2016, Tatara *et al.* 2017). A greater understanding of environmentally-induced epigenetic changes may support these efforts to improve steelhead hatchery management. Our work is among the first to demonstrate the potential for transgenerational inheritance of epigenetic information in hatchery steelhead by reporting differences in DNA methylation in the male germline. Future work should be aimed at understanding if and how these DNA methylation changes are correlated with gene expression patterns and phenotype, how these observed patterns are related to underlying genotype, and whether germline DNA methylation changes are transmissible across generations.
